# Prevalence of Hip Skeletal Deformity in Japanese Male Collegiate Long-Distance Runners

**DOI:** 10.1055/a-2898-7378

**Published:** 2026-09-02

**Authors:** Ryota Yamamoto, Toshiharu Tsutsui, Suguru Torii

**Affiliations:** 1 Graduate School of Sport Sciences 117107 Waseda University, TokorozawaTokorozawaSaitamaJapan; 2 Faculty of Sport SciencesWaseda UniversityTokorozawaSaitamaJapan

**Keywords:** runner, development, cam morphology, hip joints

## Abstract

This study aimed to examine whether hip deformity has already developed in Japanese male freshman collegiate long-distance runners and to investigate the association with their sports history.A total of 57 elite freshman collegiate long-distance runners participated. We collected sports history data during adolescence using a questionnaire, and hip images were measured using dual-energy X-ray absorptiometry. Cam morphology was defined based on visual scores of the anterior head–neck junction, classified as follows: (i) normal, (ii) flattening, and (iii) prominence. Pincer morphology was defined as a bony prominence or osteophyte-like protrusion at the acetabular rim.Cam morphology based on visual scores was found in 78 of 114 hips (68.4%; 40 right and 38 left). Pincer morphology based on visual scores was found in 73 of 114 hips (64.0%; 39 right and 34 left). There was no significant association between hip deformity and sports participation history ( *p* >0.58). In an additional exploratory non-athletic cohort, a similarly high prevalence of cam and pincer morphology was observed (cam: 14 of 18 hips [77.8%]; pincer: 13 of 18 hips [72.2%]). These may indicate that factors beyond participation in multidirectional sports, including internal factors, could contribute to hip morphology.

## Introduction

Hip/groin injury in long-distance runners account for a relatively small proportion (<20%) of running-related injuries (RRIs). 1​ In this context, RRIs generally refer to musculoskeletal conditions associated with running that result in pain, training restriction, or medical attention. However, some studies have reported a rising trend of this hip/groin injury. 2​ ​3​ Therefore, it is necessary to examine the factors contributing to hip/groin injury in long-distance runners.

Bone deformities, such as cam and pincer morphology, are internal factors that increase the risk of hip/groin injuries. 4​ 5​ ​6​ These deformities can lead to femoroacetabular impingement, a condition in which abnormal contact between the femoral head–neck junction and the acetabular rim occurs during hip motion, resulting in pain and potential joint damage. Furthermore, these deformities increase the risk of hip osteoarthritis by 4.4 to 6.2 times, 6​ ​7​ making this issue significant not only for preventing RRIs but also for ensuring the health of athletes after retirement. Outside Japan, the prevalence of bone deformity—especially cam morphology—is 60% to 80% in male athletes participating in multidirectional sports such as soccer but only 15% to 20% in male-long-distance runners, which is relatively low compared to multidirectional sports. 8​ Given this marked contrast, and considering potential ethnic differences in skeletal morphology, it remains unclear whether this pattern also applies to Japanese populations. Therefore, the present study aimed to investigate whether this relationship holds true for Japanese male long-distance runners. In addition, if an athlete has a history of engaging in multidirectional sports before transitioning to long-distance running, the presence of cam morphology may increases their risk of developing hip/groin injuries related to RRIs. Therefore, it is important to identify the hip morphology of long-distance runners to better understand and address the risks of hip/groin injuries as well as hip osteoarthritis.

The timing and underlying mechanism of hip deformity remain unclear. Most cases are believed to arise during participation in multidirectional sports during growth, often before the closure of the proximal femoral growth plate. 9​ ​10​ However, cam morphology development even after growth plate closure has been reported in a few reports. Therefore, examining long-distance runners around the typical age of closure of the proximal femoral growth plate is important for distinguishing deformities that develop during the growth period from those that arise after skeletal maturation. In this context, it is essential to determine whether hip morphotypes are already established in collegiate freshman long-distance runners, a cohort in which skeletal maturation is nearly complete. To this end, this study aimed to examine the associations between these morphological features, the athletes’ prior sports backgrounds, and the age at initiation of long-distance running.

## Methods

### Participants

For this study, all 57-freshman collegiate male long-distance runners who had competed at the national level in high school track and field competitions in Japan were included. All measurements were conducted within 2 months of enrollment to minimize the influence of college-level training. Demographic data are presented in Table 1 .

**Table:** **Table 1** Characteristics of participants with and without multidirectional sports experience

	Multi-directional sports experience		*p* value
	Yes ( *n* =40)	No ( *n* =17)	
Age (year)	18.1±0.03	18.1±0.08	0.450
Height (cm)	172.0±0.9	171.5±1.4	0.770
Weight (kg)	58.1±0.7	56.2±0.9	0.120
long-distance experience (year)	5.9±0.2	6.8±0.3	0.018*

Note: Data are presented as mean±standard deviation.

*Statistically significant difference ( *p* <0.05).

### Imaging the hip

**Fig. 1:**
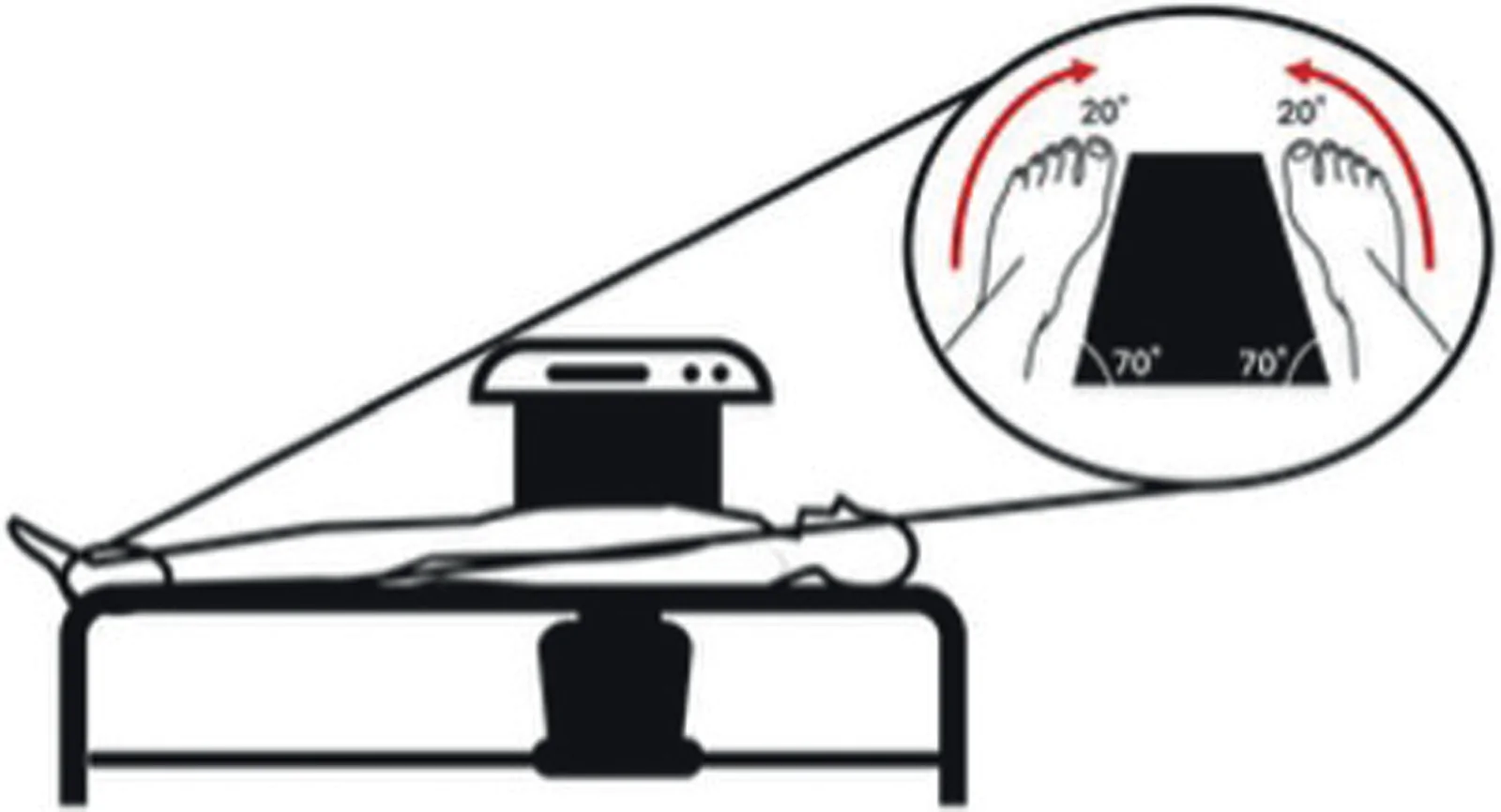
During DXA measurements, the lower limbs were positioned and secured in hip internal rotation.

Anteroposterior views of the bilateral hip joints were acquired using the hip mode of dual-energy X-ray absorptiometry (DXA; Horizon-QDR, Hologic, Bedford, MA, USA). This mode provides clearer visualization of hip morphology compared with the standard whole-body mode. Participants were positioned supine, with the pelvis aligned parallel to the measurement table and the feet secured in the manufacturer’s default fixation device to ensure that the hip joints were internally rotated ( Fig. 1 ).

Cam morphology has been assessed using X-ray and computed tomographic (CT) images. 9​ 10​ ​11​ However, recent study has validated the reliability of hip morphology measurements derived from DXA images compared to standard pelvic radiographs. In addition, previous studies, including our own, have demonstrated that DXA imaging provides a valid approach for assessing hip morphology across different age groups and sports type. 11​ 12​ ​13​ Accordingly, DXA has been increasingly used for hip morphology assessment in athlete-based and large-scale studies. In addition to its validated utility, DXA offers a substantial advantage in terms of radiation exposure (0.36–70 µSv), which is markedly lower than that of conventional hip or pelvic radiographs (600–700 µSv). 14​

### Quantification of hip deformity

Although the α-angle, defined as a measure of the sphericity of the femoral head–neck junction, is commonly used to quantify cam morphology, its assessment in the developing hip can be challenging due to the oval, rather than circular, shape of the femoral head. Consequently, previous studies have relied on visual evaluation to assess cam morphology. 9​ ​10​ In addition, Cam morphology is a recognized risk factor for osteoarthritis, and statistical shape modeling has visually demonstrated these risk morphologies. 7​ This visual scoring system has also been applied in our previous studies across various age groups and sports type, demonstrating its feasibility and consistency. 11​ ​12​ Therefore, in this study, cam morphology was assessed using the visual scoring system and definition established by Agricola et al. 10​

### Visual scores and definition of cam morphology

1. Normal: Slight symmetric concavities of the anterior head–neck junction relative to the posterior head–neck junction.
2. Flattening: Moderate decrease in the anterior head–neck offset relative to the posterior head–neck junction.
3. Prominence: Convexity in the anterior head–neck junction, in contrast to the expected concavity.

**Fig. 2:**
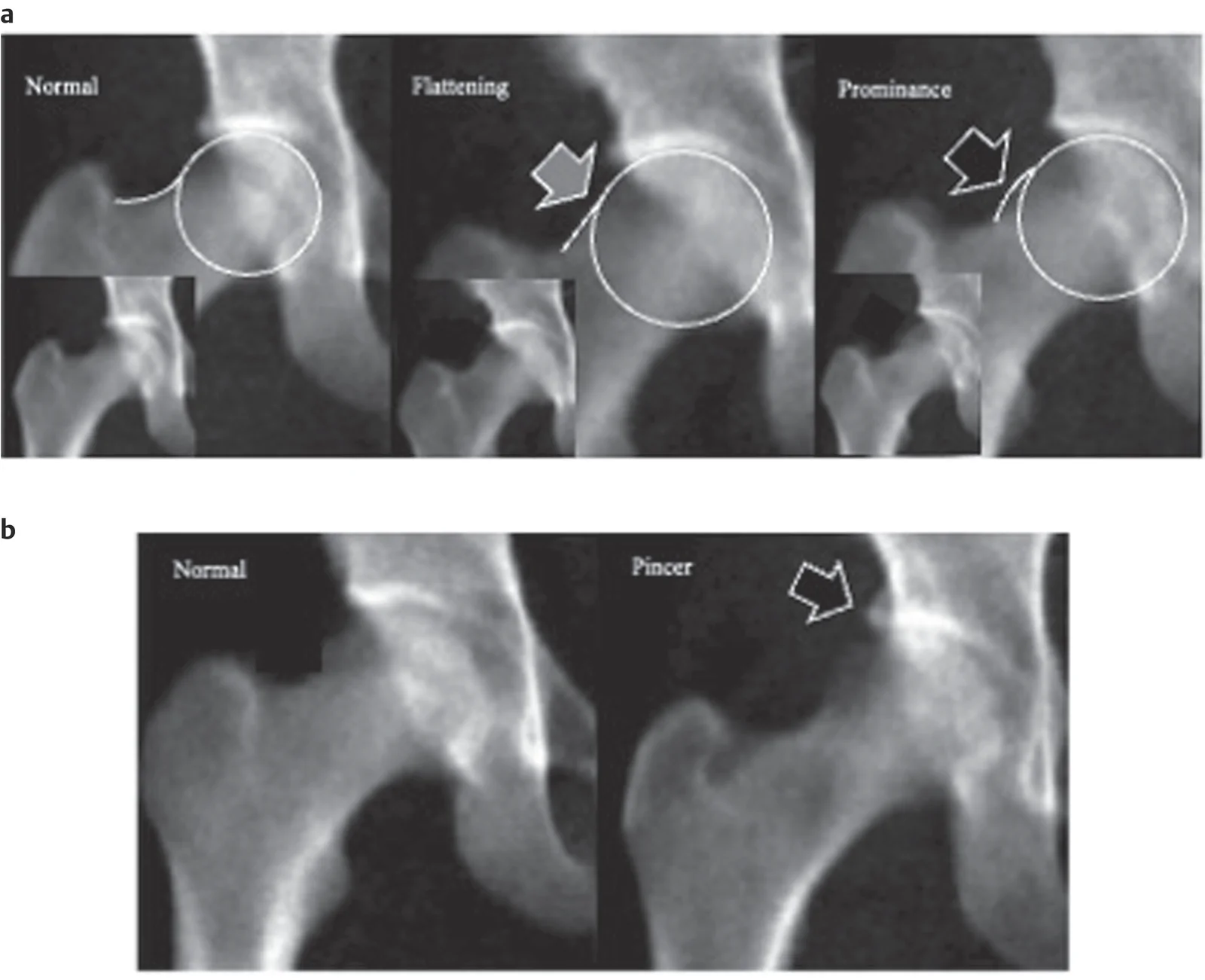
Definition of cam and pincer morphology on the DXA image. ( **a** ) The definition of visual evaluation of cam morphology. ( **b** ) The definition of visual evaluation of pincer morphology. Representative images from the study cohort are shown.

The interobserver reliability of these visual scores, assessed by very experienced orthopedic surgeons and researchers specializing in the hip joint, showed a Kappa value of 0.67. ( Fig. 2 )

### Definition of pincer morphology

A bony prominence or osteophyte-like protrusion at the rim of the acetabulum.

### Sports participation history

Participants were interviewed by team staff members regarding their sports participation history, including the types of sports played from elementary to high school and the timing of their initiation into long-distance running (>800 m). This information was then complied for analysis. Based on previous research, multidirectional sports (e.g., soccer, basketball, and volleyball) were defined as those requiring linear movement, acceleration/deceleration, changes in motion, jumping, cutting, and lateral movements during competition. 15​ 16​ ​17​ If a participant engaged in both multidirectional and other types of sports, they were classified as participating in multidirectional sports. Participants were also asked to specify when they began long-distance running, selecting from elementary school, junior high school, or high school. 18​

### Supplementary non-athletic cohort for comparison with long-distance runners

Additionally, a supplementary non-athletic cohort consisting of nine male high school students (18 hips) was recruited from individuals engaged in cultural (non-athletic) extracurricular activities in Japan. This cohort was considered to have minimal exposure to structured athletic activity. Demographic data are presented in **Supplementary Table S1 (available in the online version only)** , and representative images of hip deformities are shown in **Supplementary Fig. S1 (available in the online version only)** .

### Statistical analysis

A statistical power analysis was conducted for sample size estimation. We calculated that a minimum of 57 players in the cam morphology was needed to achieve 90% power and an alpha of 0.05.

Continuous variables are presented as mean with standard deviation (SD). The prevalence of cam morphology was described separately for the right and left legs. Fisher’s exact probability tests were used to examine associations between bony deformities and participation in multidirectional sports, as well as the timing of long-distance running initiation. A *p* -value of<0.05 was considered statistically significant. This study was designed as an exploratory observational study. A formal a priori sample size calculation was not performed because of the limited availability of prior data regarding hip morphology in freshman college long-distance runners. Statistical analyses were performed using SPSS version 27.0 for Windows.

## Results

### Participants' characteristics

The demographic data of the participants are summarized in Table 1 . Participants with and without multidirectional sports experience showed no significant differences in age, height, or weight ( *p* >0.05). However, participants without multidirectional sports experience had significantly longer long-distance running experience (6.8±0.3 y vs. 5.9±0.2 y, *p* =0.018).

### Prevalence of hip deformity

**Fig. 3:**
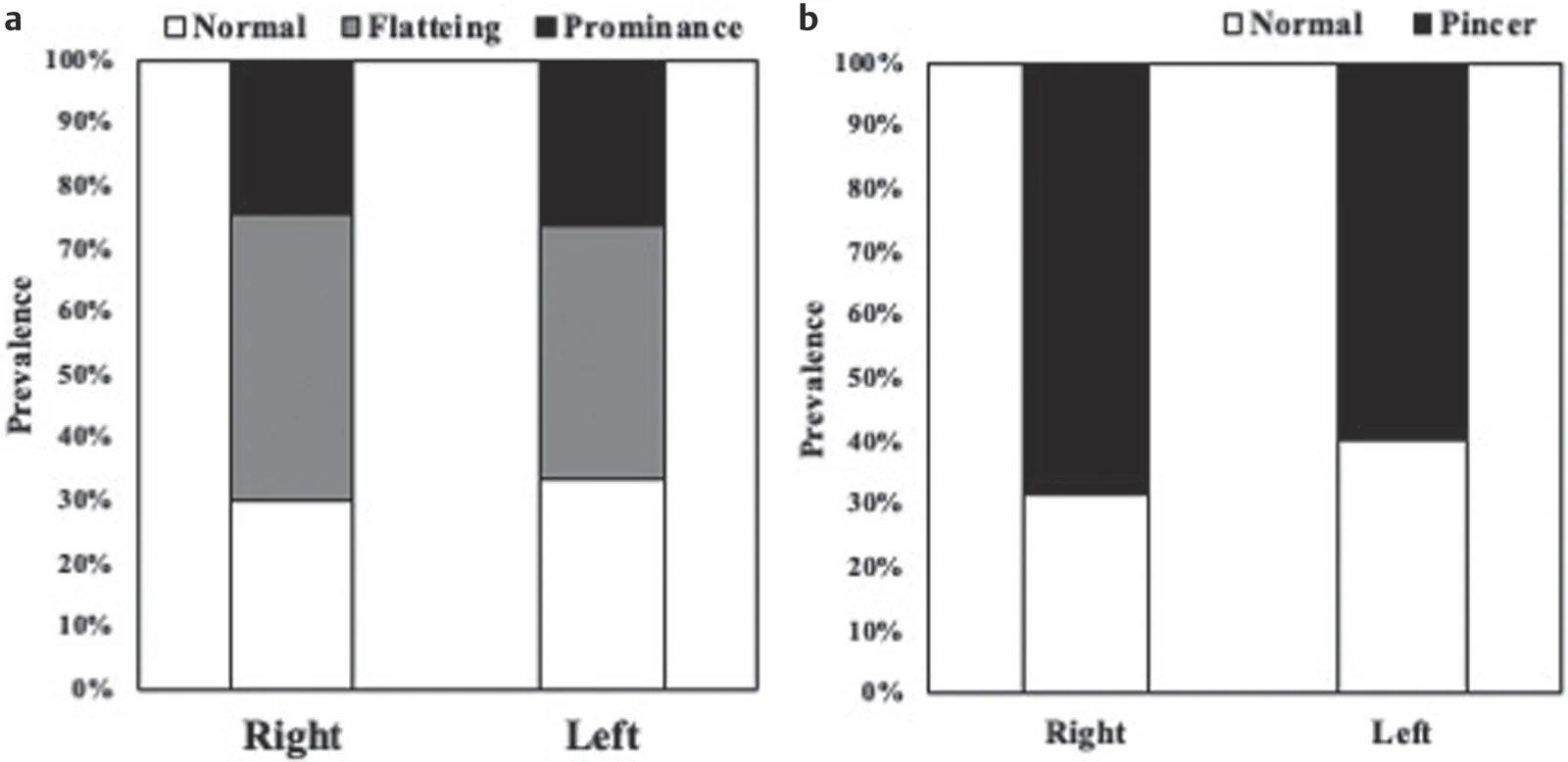
Prevalence of cam and pincer morphology in freshman college long-distance runners. ( **a** ) Prevalence of cam morphology. ( **b** ) Prevalence of pincer morphology.

Cam morphology based on visual scores was identified in 78 of 114 hips (68.4%; 40 right and 38 left). Pincer morphology based on visual scores was found in 73 of 114 hips (64.0%; 39 right and 34 left; Fig. 3 ). In an additional exploratory non-athletic cohort, cam morphology based on visual scores was observed in 14 of 18 hips (77.8%; 7 right and 7 left), and pincer morphology was identified in 13 of 18 hips (72.2%; 6 right and 7 left), providing preliminary context for the prevalence observed in the present study ( **Supplementary Table S2, available in the online version only** ).

### Sports participation history

A total of 40 participants were classified as having participated in multidirectional sports. These sports included soccer, basketball, gymnastics, badminton, and baseball. Of the remaining 14 participants, their primary involvement was in swimming events, while two participants had experience in both multidirectional sports and swimming.

### Association between history of sports participation and hip deformity

There was no significant association between cam morphology, pincer morphologies and participation in multidirectional sports for the left or right hip ( *p* >0.60; Table 2 ). Similarly, there was no association between the onset of long-distance running and hip deformity ( *p* >0.58; Table 3 ).

**Table:** **Table 2** Assocb1iation between hip deformity and prior participation in multidirectional sports

(a) Right and left cam morphology								
		Right cam morphology				df	*χ* ^2^	*p* value
		Normal	Flattening	Prominence	Total			
Multi-directional Sports experience	No	6	6	5	17	2	3.63	0.175
	Yes	11	20	9	40			
	Total	17	26	14	57			
		Left cam morphology				df	*χ* ^2^	*p* value
		Normal	Flattening	Prominence	Total			
Multi-directional Sports experience	No	7	5	5	17	2	2.75	0.247
	Yes	12	18	10	40			
	Total	19	23	15	57			
(b) Right and left pincer morphology								
		Right pincer morphology				df	*χ* ^2^	*p* value
		No	Yes	Total				
Multi-directional Sports experience	No	5	12	17		1	0.64	0.544
	Yes	13	27	40				
	Total	18	39	57				
		Left pincer morphology				df	*χ* ^2^	*p* value
		No	Yes	Total				
Multi-directional Sports experience	No	6	11	17		1	0.32	0.772
	Yes	17	23	40				
	Total	23	34	57				

*Statistically significant difference ( *p* < 0.05).

## Discussion

Although ethnic differences in skeletal structure are believed to exist, 19​ reports on hip deformities among Japanese individuals are rare. Additionally, hip deformities in long-distance runners have not been documented even in studies conducted in other countries. Previous studies have reported that the prevalence of hip deformities in long-distance athletes (15–20%) is lower than that in athletes engaged in multidirectional sports, such as soccer. 8​ In this study, the effects of past participation in multidirectional sports on cam and pincer morphology were investigated, but no significant associations were found. Notably, despite the lack of association between multidirectional sports and hip deformity, the prevalence of hip deformity among freshman collegiate long-distance runners in this study was 68.4%, which is higher than previous reports. 8​ ​20​ This prevalence is markedly higher than the 15 to 20% previously reported among long-distance runners, highlighting a clear discrepancy with the existing literature. Together with the similarly high prevalence observed in the supplementary non-athletic cohort, these findings suggest that hip deformities may develop even under relatively low levels of external mechanical loading in this population. One possible explanation is that repetitive, lower-intensity mechanical loading during skeletal development may influence hip morphology, particularly when combined with population-specific anatomical characteristics that alter the mechanical environment of the hip joint. Notably, a significant difference in long-distance running experience between groups was observed. Although no differences in hip morphology were detected using our assessment methods, cumulative exposure to long-distance running may still influence hip morphology, as running involves repetitive loading within a relatively limited range of motion, which may result in consistent stress on similar regions of the hip joint. However, these interpretations remain speculative and require further investigation.

**Table:** **Table 3** Association between hip deformity and age at initiation of long-distance running

(a) Right and left cam morphology								
		Right cam morphology				df	*χ* ^2^	*p* value
		Normal	Flattening	Prominence	Total			
Time to start long-distance running	Elementary	5	6	3	14	4	1.406	0.853
	Junior	9	17	10	36			
	High	3	3	1	7			
	Total	17	26	14	57			
		Left cam morphology				df	*χ* ^2^	*p* value
		Normal	Flattening	Prominence	Total			
Time to start long-distance running	Elementary	5	6	3	14	4	1.107	0.918
	Junior	11	14	11	36			
	High	3	3	1	7			
	Total	19	23	15	57			
(b) Right and left pincer morphology								
		Right pincer morphology				df	*χ* ^2^	*p* value
		No	Yes	Total				
Time to start long-distance running	Elementary	3	10	14		2	1.370	0.492
	Junior	13	23	36				
	High	1	6	7				
	Total	18	39	57				
		Left pincer morphology				df	*χ* ^2^	*p* value
		No	Yes	Total				
Time to start long-distance running	Elementary	4	10	14		2	1.076	0.584
	Junior	16	20	36				
	High	3	4	7				
	Total	23	34	57				

*Statistically significant difference ( *p* <0.05). Elementary: elementary school; junior: junior school; high: high school.

This study observed a symmetrical relationship between left and right hip deformities. In contrast, previous studies have demonstrated asymmetrical hip deformities in sports involving asymmetrical movement, such as soccer. 20​ The symmetry observed in this study likely reflects the symmetrical biomechanical demands of long-distance running. This symmetrical pattern may further support the notion that the observed deformities are associated with the sport-specific loading characteristics of long-distance running, rather than asymmetrical, multidirectional movements. Notably, long-distance running is characterized as a lower-impact activity compared with high-impact, multi-directional sports such as soccer and basketball; 17​ ​18​ consequently, the risk of developing cam or pincer morphology in runners has traditionally been considered low. However, a similarly high prevalence of cam and pincer morphology was also observed in an additional exploratory non-athletic cohort, despite minimal exposure to structured athletic activity. In contrast, the high prevalence identified in the present study suggests that internal factors specific to the Japanese population may play a more prominent role than the type of athletic loading alone. A previous study comparing hip deformities in elite soccer players by ethnicity reported a lower prevalence among East Asians than among White and Black athletes. 19​ However, studies focusing on Japanese populations have reported a substantially higher prevalence, contrasting with these general findings for East Asian groups suggesting potential heterogeneity within East Asian populations.

Moreover, hip joint morphology differs between Japanese and multiethnic populations, with a higher prevalence of acetabular dysplasia and a narrower acetabular cap in Japanese individuals compared to those in other countries. 21​ ​22​ In hips with mild acetabular dysplasia, the static load on the anterior acetabular cartilage is reportedly greater due to the reduced contact area between the femur and acetabulum. 23​ This results in excessive mechanical stress on the acetabulum, potentially leading to pincer morphology caused by abnormal bone formation.

Cam and pincer morphologies are now widely understood as growth-related phenomena that develop during skeletal maturation. The occurrence of cam morphology in younger individuals has recently attracted significant attention in sports science, leading to prevalence studies across various sports. In this study, the prevalence of pincer morphology among participants was 64%. Compared to previous studies, this prevalence is similar to the 70% reported in soccer players of the same age group but significantly higher than the 30% in track and field athletes. 20​ This prevalence appears closer to that reported in sports such as soccer, where bone deformities are more common; however, the underlying factors remain unclear and warrant further investigation. Importantly, these findings suggest that cam morphology in the Japanese population may occur regardless of participation in sports activities. In the muscle-skeletal development during adolesce is influenced by physical activity level and physical impact. 24​ However, recent trends indicate a decline in physical activity among Japanese adolescents, with significant differences compared to other countries. 25​ 26​ ​27​ This suggests that both internal and external factors may contribute to the high prevalence of cam morphology—even in long-distance runners, a group typically considered to have a low incidence of such morphologies. Previous studies have demonstrated that reduced mechanical loading is associated with a narrower acetabular width, 28​ suggesting that normal pelvic development is supported by appropriate mechanical stimulation. In this context, it is possible that reduced physical activity, which has been increasingly observed among Japanese adolescents, may interact with intrinsic anatomical characteristics and contribute to the high prevalence of hip deformities observed even in non-athletic individuals and long-distance runners. These findings raise the possibility that both internal and external factors jointly influence hip morphology in this population. However, these interpretations remain speculative, and further studies are needed to examine the interplay between population-specific characteristics and changes in physical activity patterns.

Several limitations of this study need to be acknowledged. First, a formal control group of non–long-distance runners or non-athletic individuals was not included in the primary analysis; however, we conducted an additional exploratory analysis using a small age-matched non-athletic cohort. While this supplementary comparison provides preliminary context for interpreting the findings, direct and definitive comparisons of hip morphology between long-distance runners and the general population remain limited. This limitation may affect the interpretation of the high prevalence observed in this study, as well as the hypotheses regarding potential population-specific factors. Future studies including appropriate control groups are needed to validate these findings and clarify the underlying mechanisms. Second, DXA images, due to their two-dimensional nature, may not always capture the site of maximum deformation. For pincer morphology, both anteroposterior and lateral views are recommended. In contrast, CT and magnetic resonance imaging provide three-dimensional assessment and may offer a more comprehensive evaluation of hip morphology. Third, a significant difference in long-distance running experience between groups was observed. This difference may reflect variations in cumulative mechanical loading associated with sport-specific activity, which could potentially influence hip morphology. However, no corresponding differences in hip morphology were detected using our assessment methods, and the impact of this factor could not be directly evaluated. Therefore, the potential confounding effect of long-distance running experience cannot be excluded. Fourth, the interobserver reliability of the visual scoring system was moderate ( *κ* =0.67), indicating a potential for variability in the assessment of hip morphology. This level of agreement may have influenced the classification of cam and pincer morphology, and the findings should therefore be interpreted with appropriate caution. Fifth, a formal a priori sample size calculation was not performed due to the limited availability of prior data regarding hip morphology in this population. As a result, the statistical power of this study may be limited, and the generalizability of the findings should be interpreted with caution. Another limitation of this study is that the study population was restricted to male freshman collegiate long-distance runners. Given the known sex-related differences in the prevalence of cam morphology, 8​ the generalizability of these findings to women may be limited. Finally, as this is a cross-sectional study with retrospective assessment of sports activity history, there is a possibility of recall bias. Therefore, prospective studies are needed to more accurately evaluate the relationship between sports participation and hip morphology. In conclusion, our study using DXA imaging found that cam morphology was present in 68.4% and pincer morphology in 64.0% of freshman college male long-distance runners. However, no association was observed between hip deformity and participation in multidirectional sports.

## Supplementary Material

Supplementary Material Zusatzmaterial
