# Supplementary material for: Prevalence of Hip Skeletal Deformity in Japanese Male Collegiate Long-Distance Runners
Source: Sports Med Int Open. 2026 Sep 2;10:a28987378. doi: 10.1055/a-2898-7378 (PMC13551460; doi:10.1055/a-2898-7378)

Figure S1.

Representative images of cam and pincer morphology in non-athletic cohort.

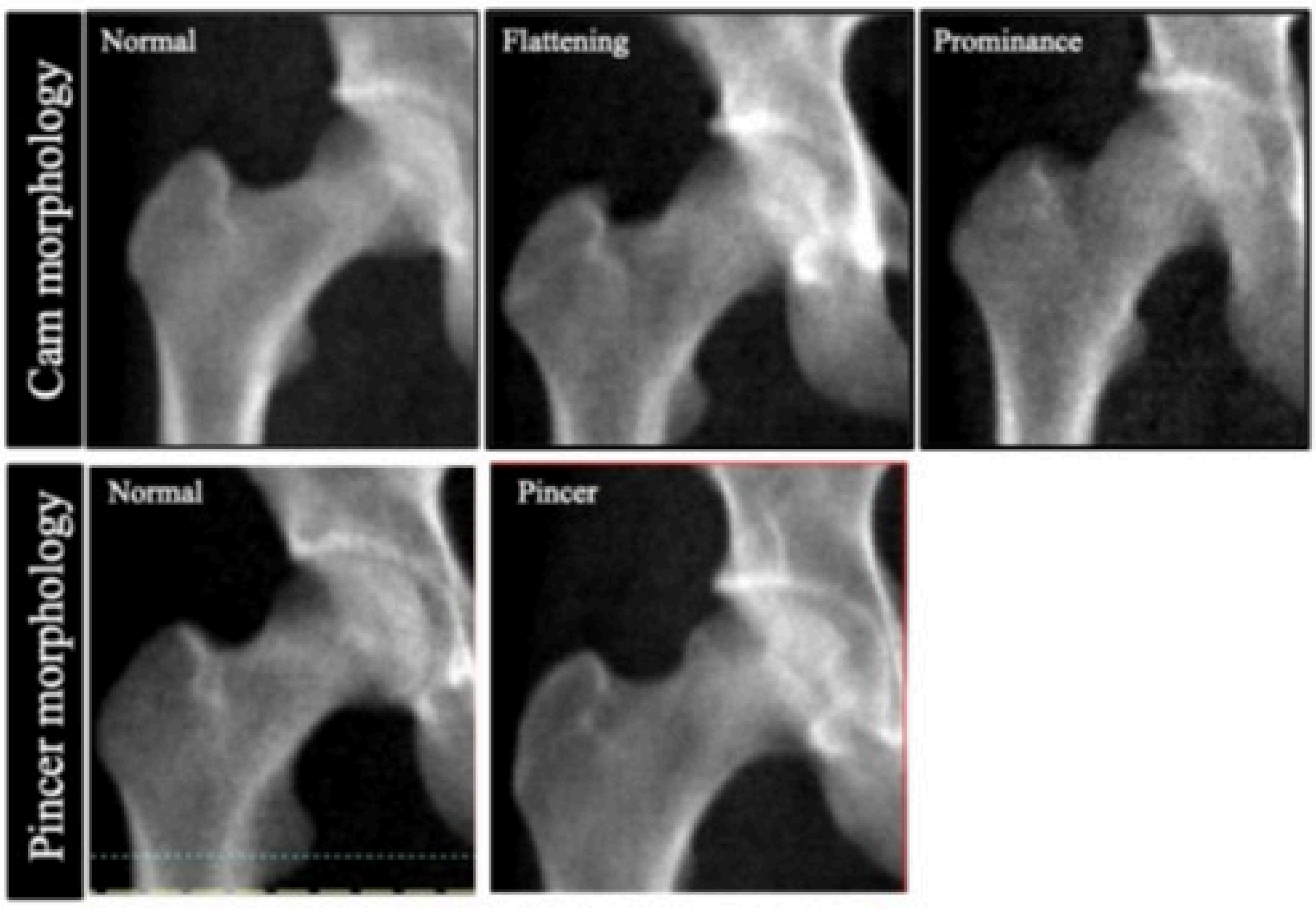

Supplement: Supplementary file 1 [file 10-1055-a-2898-7378_29358342.pdf]
